# MU-LOC: A Machine-Learning Method for Predicting Mitochondrially Localized Proteins in Plants

**DOI:** 10.3389/fpls.2018.00634

**Published:** 2018-05-23

**Authors:** Ning Zhang, R. S. P. Rao, Fernanda Salvato, Jesper F. Havelund, Ian M. Møller, Jay J. Thelen, Dong Xu

**Affiliations:** ^1^Informatics Institute, University of Missouri, Columbia, MO, United States; ^2^Christopher S. Bond Life Sciences Center, University of Missouri, Columbia, MO, United States; ^3^Department of Biochemistry, University of Missouri, Columbia, MO, United States; ^4^Department of Molecular Biology and Genetics, Aarhus University, Aarhus, Denmark; ^5^Department of Electrical Engineering and Computer Science, University of Missouri, Columbia, MO, United States

**Keywords:** machine learning, mitochondrial targeting, deep neural network, support vector machine, position weight matrix, gene co-expression

## Abstract

Targeting and translocation of proteins to the appropriate subcellular compartments are crucial for cell organization and function. Newly synthesized proteins are transported to mitochondria with the assistance of complex targeting sequences containing either an N-terminal pre-sequence or a multitude of internal signals. Compared with experimental approaches, computational predictions provide an efficient way to infer subcellular localization of a protein. However, it is still challenging to predict plant mitochondrially localized proteins accurately due to various limitations. Consequently, the performance of current tools can be improved with new data and new machine-learning methods. We present MU-LOC, a novel computational approach for large-scale prediction of plant mitochondrial proteins. We collected a comprehensive dataset of plant subcellular localization, extracted features including amino acid composition, protein position weight matrix, and gene co-expression information, and trained predictors using deep neural network and support vector machine. Benchmarked on two independent datasets, MU-LOC achieved substantial improvements over six state-of-the-art tools for plant mitochondrial targeting prediction. In addition, MU-LOC has the advantage of predicting plant mitochondrial proteins either possessing or lacking N-terminal pre-sequences. We applied MU-LOC to predict candidate mitochondrial proteins for the whole proteome of Arabidopsis and potato. MU-LOC is publicly available at http://mu-loc.org.

## Introduction

Mitochondria play an essential role in plant cells. They are responsible for a diversity of biological processes, such as energy production, biosynthesis of several co-factors and vitamins, photorespiration, and programmed cell death (Millar et al., 2011; Welchen et al., 2014). It has been estimated that more than 95% of mitochondrial proteins in plants are encoded by nuclear genes (Millar et al., 2005). Two basic mechanisms exist for mitochondrial targeting. One group of proteins has cleavable N-terminal targeting peptides (also called pre-sequences), and the other group does not possess pre-sequences and instead has short, internal signal sequences that are still not well characterized (Chacinska et al., 2009; Schmidt et al., 2010). Additionally, there are reports of “piggyback” protein import and “sharing” of proteins through connections with the endoplasmic reticulum (ER), though these are exceptions to primary routes of import (Badugu et al., 2008; Fransen et al., 2012).

A great effort has been made to identify plant mitochondrial proteins at the whole proteome level. Mass spectrometry and fluorescence-tagging techniques are the most frequently used experimental methods to determine protein subcellular localization. To date, the highest number of experimentally identified mitochondrial proteins from a single study in plants is 1,060 from potato tubers (Salvato et al., 2014). Also, the Uniprot/Swiss-Prot database (release 2016_08) contains 830 annotated Arabidopsis proteins with curated mitochondrial localization annotations. However, it is estimated that the Arabidopsis proteome contains about 2,000–3,000 mitochondrial proteins (Millar et al., 2005, 2006; Hooper et al., 2014). Therefore, plant mitochondrial proteomes identified to date are far from complete. Despite the development of high-throughput technologies, identifying plant mitochondrial proteins experimentally is still a time-consuming and labor-intensive task. In addition, mass spectrometry has limitations in discovering a wide variety of “extreme” proteins including low mass, low abundance, or highly hydrophobic proteins. Computational methods, on the other hand, provide an efficient and cost-effective large-scale method to predict plant mitochondrial proteins.

A number of computational tools have been developed to predict mitochondrial proteins. These tools apply a diversity of prediction algorithms, such as expert system, discriminant analysis, decision tree, k-nearest neighbor (KNN), neural network, and support vector machine (SVM; Rao et al., 2016). Most of the programs use the N-terminal sequence information in their predictions. TargetP is one of the most frequently used tools for mitochondrial localization prediction. It builds multiple neural networks for different subcellular locations and then combines them using an integrating network (Emanuelsson et al., 2000, 2007). Predotar is also a neural network-based program to identify N-terminal targeting sequences (Small et al., 2004). It utilizes amino acid composition, charge, and hydrophobicity information as input features. MitoProt II uses a discriminant analysis to predict proteins imported to mitochondria and their N-terminal targeting signals (Claros and Vincens, 1996). WoLF PSORT employs sorting signals, amino acid composition, and functional motif information to train a KNN classifier for prediction (Horton et al., 2007). MitoFates is a relatively new method incorporating amino acid composition, physico-chemical information, amphiphilic property of pre-sequences, position weight matrices, and sequence motifs in an SVM model to predict mitochondrial N-terminal pre-sequences (Fukasawa et al., 2015). LOCALIZER is a recent machine-learning method for predicting localizations of mitochondria, chloroplasts, and nuclei proteins in plants (Sperschneider et al., 2017). DeepLoc uses recurrent neural network (RNN), a deep learning architecture, to predict 10 subcellular locations including mitochondria (Almagro Armenteros et al., 2017). However, unlike other tools, DeepLoc does not have a separate model specifically for plants as plant proteins may have different patterns of targeting signals compared with animals and fungi. Meanwhile, several studies have explored gene and protein functional information as training features. SUBAcon integrates 22 prediction tools and four types of experimental evidences including protein–protein interactions and gene co-expression in a Bayesian classifier to predict the subcellular localizations for all proteins in Arabidopsis (Hooper et al., 2014). YLoc, an interpretable protein targeting prediction system, utilizes gene ontology information in its prediction (Briesemeister et al., 2010). Cui et al. (2011) also released a predicted mitochondrial proteome of 2,585 proteins for Arabidopsis using gene co-expression information.

Despite various computational methods available, current tools have relatively poor performance when applied to predict mitochondrial proteins in plants. Salvato et al. (2014) pointed out that current methods failed to predict approximately 35% of the Arabidopsis and potato mitochondrial proteomes. First, most of these programs were developed years ago and relied on the limited data available at that time. Nowadays, with more experimentally identified plant mitochondrial proteins, we have more enriched training data at hand. Second, most tools focus on features from N-terminal pre-sequences. However, the N-terminal targeting is only one of import mechanisms of mitochondrial proteins (Calvo and Mootha, 2010). Third, most tools employ traditional machine-learning methods. In recent years, deep learning has been successfully used to solve various biological problems and has shown superiority over traditional machine-learning algorithms (Angermueller et al., 2016; Rampasek and Goldenberg, 2016; Almagro Armenteros et al., 2017). To the best of our knowledge, applying deep learning approaches to answer the question of mitochondrial localization in plants is novel, and we here show that out method leads to improved prediction performance.

To address the limitations of current methods, we developed MU-LOC, a machine-learning framework for plant mitochondrial protein prediction. Our contributions to the field include (1) collecting a comprehensive and high-quality dataset of mitochondrial proteins currently available in plants; (2) combining functional features in addition to N-terminal sequence information, including amino acid compositions, protein position weight matrix, and gene co-expression; (3) taking advantage of machine-learning methods [deep neural network (DNN) and SVM] to achieve a substantial improvement over existing tools; and (4) having the capacity of predicting plant mitochondrial proteins with and without N-terminal pre-sequences. MU-LOC is publicly accessible at http://mu-loc.org.

## Materials and Methods

The problem of predicting plant mitochondrial proteins can be viewed as a binary classification problem: given a plant protein, we would like to classify it as a mitochondrial or non-mitochondrial protein. Machine-learning methods are suitable for addressing this problem. There are several key steps in this process: (1) constructing a high-quality labeled dataset; (2) extracting a set of features with effective discrimination power for the two categories; (3) developing a training classifier; and (4) testing the prediction performance of the classifier. We discuss each step in the following sections.

### Data Collection and Pre-processing

**Table 1** summarizes the datasets we used in this study. We have used a set of experimentally validated mitochondrial proteins in plants reported by several high-throughput researches (Heazlewood et al., 2004; Carrie et al., 2009; Huang et al., 2009; Duncan et al., 2011; Tan et al., 2012). We obtained from the literature 541, 327, and 1,060 mitochondrial proteins for Arabidopsis, rice, and potato, respectively. It should be noted that these high-throughput experiments might include certain false-positive results, but the error rate is believed to be small and should not have significant effects on the prediction model.

**Table 1:** Plant mitochondrial protein data collected in this study.

Class	Data source	Species	Number of proteins	Reference
Positive (1,104)	Literature	*Solanum tuberosum*	1,060	Salvato et al., 2014
	Literature	*Arabidopsis thaliana*	541	Heazlewood et al., 2004; Carrie et al., 2009; Huang et al., 2009; Duncan et al., 2011; Tan et al., 2012
	Literature	*Oryza sativa*	327	Huang et al., 2009
	PPDB	*A. thaliana*	460	Sun et al., 2009
	PPDB	*Zea mays*	666	Sun et al., 2009
	SUBA3	*A. thaliana*	1,196	Tanz et al., 2013
	Uniprot/Swiss-Prot	Multiple plants^a^	1,547	UniprotKB
Negative (5,809)	Uniprot/Swiss-Prot	Multiple plants^a^	27,966	UniprotKB

*Numbers in parentheses represent proteins after running the redundancy reduction procedure. ^*a*^Supplementary Table S1 lists the top 15 species distribution of the Uniprot/Swiss-Prot datasets.*

As a secondary data source, we have also collected data from several public databases under rigorous standards. UniProt/Swiss-Prot database has protein subcellular localization annotations (The UniProt Consortium, 2015). We downloaded the Swiss-Prot flat files (release 2016_08) and extracted manually asserted mitochondrial proteins belonging to Viridiplantae based on NCBI Taxonomy. In this way, 1,547 plant mitochondrial proteins were retrieved. Supplementary Table S1 lists the top 15 species with the highest number of mitochondrial targeting annotation in Uniprot/Swiss-Prot. The Plant Proteome Database (PPDB) contains manually curated subcellular localizations for Arabidopsis and maize proteins (Sun et al., 2009). We downloaded the sequences of 460 and 666 mitochondrial proteins for *Arabidopsis thaliana* and *Zea mays*, respectively. SUBA3 is a subcellular localization database for Arabidopsis proteins, from which we retrieved 1,196 mitochondrial proteins experimentally determined by GFP or MS/MS (Tanz et al., 2013). On the other hand, the negative dataset was constructed from the Uniprot/Swiss-Prot database with evidence of manual assertions. In total, 27,966 non-mitochondrial proteins in plants were extracted and treated as the negative set. Supplementary Table S1 lists top 15 species most frequently used in the negative data. Some proteins in the negative set might be mis-annotated and actually be mitochondrial proteins, but again its impact on the prediction model is believed to be small. On our website^1^, we listed the detailed information of the Swiss-Prot dataset, including the protein ID, its description, subcellular localization annotation, species, and targeting signals if available.

After combining all positive and negative protein sequences, a non-redundant dataset was constructed using CD-HIT (version 4.6; Li and Godzik, 2006; Huang et al., 2010) with a sequence identity threshold of 40%. Similar proteins were first grouped into different clusters. The longest protein in each cluster was chosen as the representative sequence. Also, sequences with less than 50 amino acids were excluded. Finally, we obtained 1,104 and 5,809 non-redundant proteins as the positive and negative sets, respectively.

We further partitioned the data collected into training, specificity estimation (validation), and independent testing set. Supplementary Table S2 lists the number of proteins in each subset, where 1,000 proteins from both positive and negative set were randomly selected to construct the training set. Meanwhile, additional 4,500 proteins from the negative set were used for specificity estimation. In practice, this gives a confidence level measure when classifying unknown proteins. Furthermore, 100 proteins each from both positive and negative sets not used in training or specificity estimation were set aside as an independent testing set to compare the prediction performance of MU-LOC with existing tools. The testing set containing 100 positive cases is a mixture of mitochondrial proteins with or without N-terminal pre-sequences, reflecting the real-world use case where we do not know if a mitochondrial protein has an N-terminal targeting sequence or not.

We included a second independent testing set which Sperschneider et al. (2017) used, containing curated 65 plant mitochondrial and 587 non-mitochondrial proteins, respectively (Supplementary Table S2). The 65 mitochondrial proteins have N-terminal pre-sequences, and we used this dataset to evaluate the performance of MU-LOC and existing tools in predicting plant mitochondrial proteins with N-terminal pre-sequences. It should be noted that the overlap of these 652 proteins with our training data is minimal. In total, six mitochondrial and 16 non-mitochondrial proteins were in our training set.

### Feature Extraction

#### Amino Acid Frequency Features

Amino acid frequencies are commonly used features for protein subcellular localization prediction. We extracted the frequencies of the 20 types of amino acids from both N-terminal and the whole sequences, respectively. For N-terminal features, by default, we used the first 22 residues to calculate the amino acid frequency features. The feature length of amino acid frequency is 20 and the sum of the 20 frequencies is one.

#### Sequence Profile Features

Sequence profile information is another set of features that some prediction programs employ. We used the position-specific scoring matrix (PSSM) to reflect the evolutionary profile of a protein. PSI-BLAST (blastpgp and makemat, version 2.2.26) was applied to obtain raw PSSMs with the following parameter settings “-j 3 -h 0.001” (three iterations, and inclusion e-value as 0.001; Altschul et al., 1990). We used a non-redundant UniProt/Swiss-Prot database (release 2016_08) as the search database. Then, each entry in the raw PSSM was scaled linearly to the range of -1 to 1.

In a PSSM, each position of a protein sequence is represented by a vector of 20 showing the preferences of the 20 types of amino acids at this position throughout evolution. We computed PSSM features for both N-terminal and the whole sequences. For the N-terminal PSSM feature, similar to the amino acid frequency feature, we used the vector representation of the first 22 residues. Therefore, the N-terminal PSSM feature has a length of 440 (20 by 22). On the other hand, for the global PSSM feature, we averaged the vectors representing the same amino acid type. Therefore, the global PSSM feature can, to some extent, be viewed as a generalized amino acid frequency (average pooling) where each type of amino acid is represented by a vector indicating the overall usage of the 20 amino acids through evolution. By necessity, the position-specific information is eliminated here, but we obtained the global PSSM feature with a fixed dimension for proteins with varying lengths. The global PSSM has a feature length of 400 (20 by 20).

#### Gene Co-expression Features

Functional data, such as gene co-expression, captures complementary information compared with sequence features. The hypothesis is that mitochondrial genes tend to co-express with mitochondrial rather than non-mitochondrial genes to form various pathways and perform their functions. SUBAcon also utilized gene co-expression information in their prediction, which increased the Matthews correlation coefficient (MCC) value for mitochondrion targeting by 2.5% (Hooper et al., 2014). We downloaded the NCBI GEO Datasets (GDS) for *A. thaliana* with at least 10 samples and experimental platform GPL198, and obtained 24 datasets (Supplementary Table S3; Barrett et al., 2013). These 24 GEO datasets were already normalized by NCBI, and we performed log2 transformations to all gene expression values. We then calculated the Pearson’s correlation coefficient matrix for each individual dataset, and followed a meta-analysis approach by combining the 24 correlation matrices to compute pairwise meta-Pearson’s correlation coefficients (meta-PCCs) between 20,839 Arabidopsis genes (Srivastava et al., 2010). More detailed description of the meta-analysis is provided in Supplementary Materials.

We applied the idea of KNN to turn the meta-PCC co-expression matrix into input features. We mapped both training and testing proteins to Arabidopsis by BLAST (blastp, version 2.2.26; Altschul et al., 1990) against the Arabidopsis proteome (downloaded from TAIR; Lamesch et al., 2012) to find the best hit in Arabidopsis (e-value of 0.01 as cut-off). Next, for each testing protein, we extracted top *K*% genes/proteins in the training set that had the highest meta-PCCs with the query protein. Then we calculated the percentage of positive set in the *K*% most highly co-expressed genes, and denoted this percentage as the KNN score. In this way, the meta-PCC co-expression matrix was turned into a KNN score. We set *K* as 0.25, 0.5, 1, 2, and 4% of the training data to obtain five KNN scores as input features. The choice of *K* values was consistent with Gao et al. (2010) from which we borrowed the idea of KNN scores. In case we could not find matches satisfying the e-value cut-off, we assigned five zeros for the co-expression feature. The gene co-expression feature has a length of five.

### Machine Learning and Feature Selection

We applied two types of machine-learning methods in the process of feature selection and model training, including SVM and DNN.

#### Support Vector Machine (SVM)

We used the software package SVM*^light^* (version 6.02^2^) to train SVM classifiers. Radial basis function (RBF) was used as the kernel function. The best parameters were obtained using a stratified 10-fold cross-validation by searching a grid of cost and gamma values. The search range was [10^-3^, 10^-2^, 10^-1^, 1, 10, 10^2^, 10^3^] for the cost parameter (-c option) and [10^-5^, 10^-4^, 10^-3^, 10^-2^, 10^-1^, 1, 10, 10^2^, 10^3^, 10^4^, 10^5^] for the gamma parameter (-g option). As a result, we ran the program with optimal parameters of “-c 10 -g 0.1” for SVM*^light^*. We also performed stratified 10-fold cross-validation to evaluate the performance of features and training sample sizes. Briefly, the 2,000 training proteins were partitioned randomly into 10 sub-samples. Each sub-sample contained 100 positive and negative proteins, respectively. Then we iterated the process 10 times where sub-sample *i* was used as validation for testing the model and the other sub-samples as training for building the model in iteration *i* (*i* = 1, ⋅⋅⋅, 10). On the other hand, when making predictions on specificity estimation and independent testing set, all 2,000 training proteins were used to build a single prediction model.

#### Deep Neural Network (DNN)

We used the Python machine learning library Theano (version 0.7.0)^3^ and Pylearn2^4^ to build feed-forward DNN models. Due to the large number of parameters, we did not perform a comprehensive parameter optimization. Instead, we optimized some key parameters using a stratified 10-fold cross-validation, including the learning rate, number of hidden layers and hidden units, and dropout probability. Our optimal DNN consisted of three hidden layers with 500, 500, and 300 hidden units, respectively. Meanwhile, the output layer used the Softmax function. The learning rate was set to 0.01. The initial momentum was set to 0.5 and increased to 0.7 at the end of training. We also applied the Maxout (Goodfellow et al., 2013) function as the activation function, and the technique of dropout (Srivastava et al., 2014) to achieve better performance and avoid overfitting. Maxout as an activation function can approximate any convex function and is a natural companion to dropout (Goodfellow et al., 2013). The key idea of dropout is to randomly exclude a certain number of hidden units along with all the associated weights in each iteration and generate a large number of simplified sub-networks. The average weights from all the sub-networks are used at the end of each iteration. We set the Maxout components to five and the dropout probability to 0.5 for all three hidden layers. To facilitate the training process, we also took advantage of the capability of GPU computing provided by Theano. This allowed us to train a model with hundreds of thousands of parameters within 15 min.

As with SVM, we applied stratified 10-fold cross-validation to evaluate the performance of features and training sample sizes. In each iteration, eight sub-samples were used to train a DNN model, one sub-sample was treated as validation to choose the best model, and the remaining one sub-sample was used as the testing set to measure the cross-validation performance. This process was repeated 10 times and each sub-sample was used as the validation or testing set exactly once. When making predictions on specificity estimation and independent testing set, we merged the training and testing sub-samples in cross-validation, and still used the validation sub-sample to choose the best model. In this way, 10 DNN models were trained, and the final predictions were the averages of the output results from the 10 models.

#### Feature Selection

For the N-terminal features, the length of N-terminal sequences used to extract features becomes a key parameter. We extracted 704 plant mitochondrial proteins with transit peptide annotations from the Uniprot/Swiss-Prot data collected. About 70% of the 704 proteins have transit peptides with a length of 5–50 amino acids (Supplementary Figure S1A). Therefore, we computed amino acid frequency and PSSM features from 5 up to 50 N-terminal residues, respectively. We then tested the prediction performance of each individual feature using SVM and 10-fold cross-validation. The corresponding average areas under the receiver operating characteristic (ROC) curve (AUCs) were computed as performance evaluation measurements.

For features generated from whole sequences, we computed amino acid frequency, PSSM, and gene co-expression features. Similar to the N-terminal features, we tested the prediction performance of each type of feature using SVM and 10-fold cross-validation, and computed the average AUCs to evaluate the performance.

### Performance Measures

We applied several frequently used metrics to evaluate the prediction performance of our models, including accuracy, specificity, sensitivity, precision [true positive prediction percentage among all the predictions, or positive predictive value (PPV)], F_1_ score, and MCC. Supplementary Table S4 summarizes the above measurement metrics and the corresponding equations to calculate them. ROC and precision–recall curves were plotted by taking different thresholds, and AUCs were calculated using the trapezoidal rule.

### Data Availability

MU-LOC is publicly available at http://mu-loc.org as a web-service. The data and program are also publicly available for download at the website. We also created an Amazon Machine Image (AMI) hosted at Amazon Web Services (AWS). This virtual machine enables users to run MU-LOC for proteome-wide prediction without concerning about installing any prerequisite tools or data. More details about how to use the Amazon AMI are described on MU-LOC website.

## Results

### Discrimination Capability of Individual and Combined Features

We explored different types of features generated using both N-terminal and whole sequences. We performed 10-fold cross-validation on the training set for each feature combination and calculated the average AUCs as performance measurements. Both N-terminal amino acid frequency and PSSM features reached their highest average AUCs with 20–30 residues at the N-terminus (Supplementary Figure S1B). By default, features computed from the N-terminal 22 amino acids were included in the final training as N-terminal features. The N-terminal amino acid frequency and PSSM feature achieved an average AUC of 0.721, and 0.775, respectively (Supplementary Table S5). Features from whole sequences including amino acid frequency, PSSM, and gene co-expression feature had an average AUC of 0.669, 0.810, and 0.742, respectively (Supplementary Table S5). Also, adding whole sequence amino acid frequency to other features did not improve the prediction performance (Supplementary Table S5). Hence, we did not include whole sequence amino acid frequency in the final training features. To conclude, the features we finally incorporated in our training and testing were N-terminal amino acid frequency (AAFreq.NT), N-terminal PSSM (PSSM.NT), whole sequence PSSM (PSSM), and gene co-expression (Coexpr).

Supplementary Table S6 summarizes the prediction power of combinations of the final input features. Every individual feature achieved an average AUC greater than 0.72. Whole sequence PSSM performed the best and reached an average AUC of 0.810 and 0.804 using SVM and DNN, respectively. It should also be noted that using gene co-expression alone with feature length of only five, the average AUC still reached 0.742 with the SVM model. We failed to train a working DNN in 250 epochs using AAFreq.NT, PSSM.NT, and Coexpr, respectively, suggesting that DNN was more difficult to train than traditional machine-learning methods. When combining all four types of features, the discrimination power further improved to 0.850 and 0.857 for SVM and DNN models, respectively. This indicated that the four sets of features captured complementary information from different aspects. Therefore, integrating all features together worked better than any individual feature.

We also tested the prediction performance using three out of the four types of features to further investigate the impact of different features. When AAFreq.NT was excluded from training, the average AUC (0.850 for SVM and 0.855 for DNN) remained very close to the AUC with all four types of features (0.850 for SVM and 0.857 for DNN). For the other three sets of features, the performance decreased dramatically when one was removed. Without using PSSM, the performance dropped the most to 0.820 and 0.796 for SVM and DNN, respectively. Removal of Coexpr also decreased the performance substantially. When excluded from training, the average AUC dropped to 0.824 (SVM) and 0.821 (DNN). When removing PSSM.NT, the SVM model had similar performance compared with all features used while the DNN model showed a decrease of the average AUC to 0.835. This may indicate a certain level of redundancy between PSSM.NT and the other features, and some superiority of DNN compared to SVM in modeling features with redundancy. In summary, the discerning powers of different types of features in decreasing order were PSSM, Coexpr, PSSM.NT, and AAFreq.NT. In the following sections, we discuss these features in more detail.

### Amino Acid Frequency Features

**FIGURE 1:**
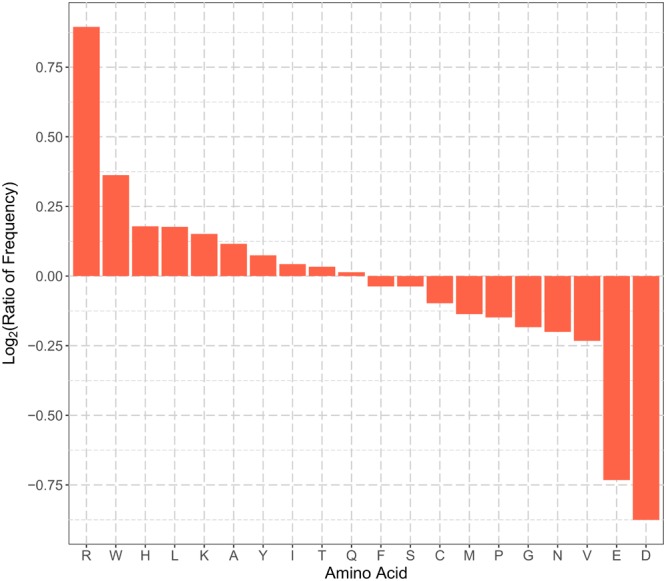
Comparisons of N-terminal sequence amino acid frequencies in the positive and negative training datasets. The vertical axis represents the log_2_ ratio of average frequency of each type of amino acid for the N-terminal 22 residues between positive and negative training set. Amino acid residues in the horizontal axis are sorted in the descending order of the log_2_ ratio. A value greater than zero indicates that the corresponding amino acid residue is enriched in the N-terminal sequences of plant mitochondrial proteins, and vice versa.

Amino acid frequency, especially the N-terminal amino acid composition, is a type of commonly used features for protein subcellular localization prediction. **Figure 1** summarizes the enrichment of the 20 types of amino acids in the N-terminal sequences (22 residues) of plant mitochondrial proteins. Arg, Trp, His, Leu, Lys, Ala, Tyr, and Ile were enriched in the N-terminal sequences of plant mitochondrial proteins. The enriched amino acids can be grouped into two categories, positively charged (Arg, His, and Lys), and hydrophobic (Trp, Leu, Ala, Tyr, and Ile). On the other hand, Asp, Glu, Val, Asn, Gly, Pro, and Met were depleted in the positive dataset. In particular, the two negatively charged residues, Asp and Glu, had the most depletion in the N-terminal sequences. This is consistent with previous studies, where pre-sequences of mitochondrial proteins were found to form amphiphilic helices with hydrophobic residues on one side and positively charged residues on the other side (von Heijne, 1986; Schneider et al., 1998). Therefore, the enrichment of positively charged and hydrophobic residues was expected. Hence, plant mitochondrial proteins and non-mitochondrial proteins had different patterns of the amino acid composition in their N-terminus, and the corresponding amino acid frequency can be used as discriminative features for predicting mitochondrial targeting.

### Sequence Profile Features

**FIGURE 2:**
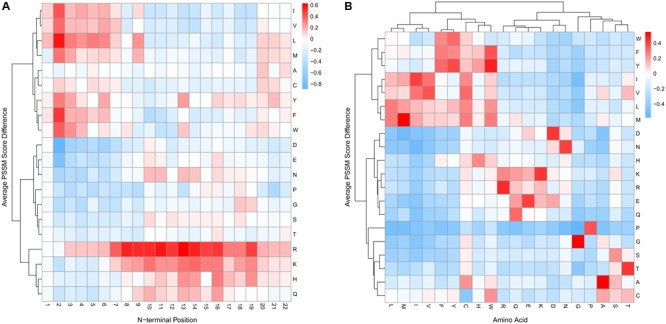
Comparisons of PSSM scores in the positive and negative training datasets. Positive values (cells in red) indicate higher average PSSM scores of mitochondrial proteins compared with non-mitochondrial proteins and vice versa for negative values (cells in blue). **(A)** The average PSSM score difference between the positive and negative set for the N-terminal 22 residues. Each column represents the difference of the 20 amino acid usages in an N-terminal position between mitochondrial and non-mitochondrial proteins. Hierarchical clustering was performed using Ward’s method. **(B)** The average PSSM score difference between mitochondrial and non-mitochondrial proteins in the training set for the whole protein sequences. Given a sequence, the PSSM scores were averaged for each type of amino acid. We then computed the difference of the averaged PSSM scores between the positive and negative set in the training data. Hierarchical clustering was also performed using Ward’s method.

**Figure 2** summarizes the PSSM features for N-terminal and whole sequences, respectively. For N-terminal PSSM features, the positive and negative sets had different amino acid usage at different sequence positions (**Figure 2A**). Hydrophobic residues (Ile, Val, Leu, Met, Ala, Tyr, Phe, and Trp) were preferred in the sequence at the very beginning of mitochondrial proteins (positions 1–6), while the positively charged residues, Arg, Lys, and His, were enriched in most N-terminal positions. On the other hand, the negatively charged residues, Asp and Glu, were depleted in most N-terminal positions. These findings are consistent with amino acid composition, but the N-terminal sequence profiles had better predictive power than amino acid frequencies (Supplementary Table S6). Hence, the N-terminal PSSM features can be included as discriminative features.

For global PSSM features, the 20 types of amino acids formed three cluster blocks (**Figure 2B**). One block mainly contained hydrophobic residues (Trp, Phe, Tyr, Ile, Val, Leu, and Met). Another block mainly contained polar and charged residues, including Asp, Asn, His, Lys, Arg, Glu, and Gln. In addition, the positive set generally had larger PSSM scores within each block (**Figure 2B**, cells in red). This indicated that mitochondrial proteins might prefer to use amino acids with similar properties (hydrophobic, charged, polar, etc.) compared with non-mitochondrial proteins, and thus tended to be conserved. Hence, the global sequence profile information was also a useful discriminative feature. In addition, from the previous section, we see that the global PSSM features had the best discriminative power (Supplementary Table S6).

### Gene Co-expression Features

**FIGURE 3:**
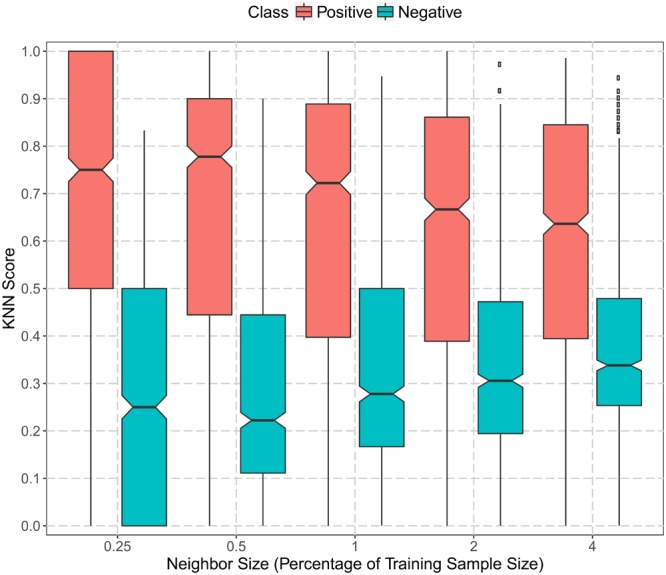
Boxplot of gene co-expression KNN scores in the positive and negative training datasets. The vertical axis represents the KNN scores computed as the proportion of the positive set in the nearest neighbors (genes in the training set with the top ranked meta-Pearson’s correlation coefficients). The horizontal axis denotes the size of the nearest neighbors as the percentage of training sample size (0.25, 0.5, 1, 2, and 4%). The black dots represent data points beyond the upper quartile.

We applied KNN score to utilize the gene co-expression information. KNN score was calculated as the percentage of the positive set in *K*% nearest neighbors that had the highest meta-PCCs. The larger the score, the more similar a protein was to mitochondrial proteins. **Figure 3** summarizes the KNN score with different parameter settings of *K* (0.25, 0.5, 1, 2, and 4%). Overall, there was a clear separation of the KNN scores for positive and negative datasets. Mitochondrial proteins had a median KNN score greater than 0.6. On the other hand, non-mitochondrial proteins had a median KNN score less than 0.4 and the 75% quantile was less than 0.5. Note that this separation was not due to sequence similarity since the redundancy had already been reduced to less than 40% for the collected data. This indicates that plant mitochondrial proteins were, to some extent, conserved with respect to their functions and expression. Positive proteins tended to be co-expressed with mitochondrial proteins, while negative proteins were less likely to be co-expressed with mitochondrial genes. Therefore, the functional gene co-expression information and the KNN score representations were suitable as discriminative features for mitochondrial targeting prediction.

### Effect of Training Sample Size

To evaluate the effect of different training sample sizes, we randomly selected an *x*% (*x* = 50, 60, ⋅⋅⋅, 100) subset from the 2,000 training samples. Each subset contained an equal number of positive and negative proteins. We extracted all four types of features, namely, AAFreq.NT, PSSM.NT, PSSM, and Coexpr. Both SVM and DNN models were trained with 10-fold cross-validation, and the average AUCs were calculated as performance measurements.

**FIGURE 4:**
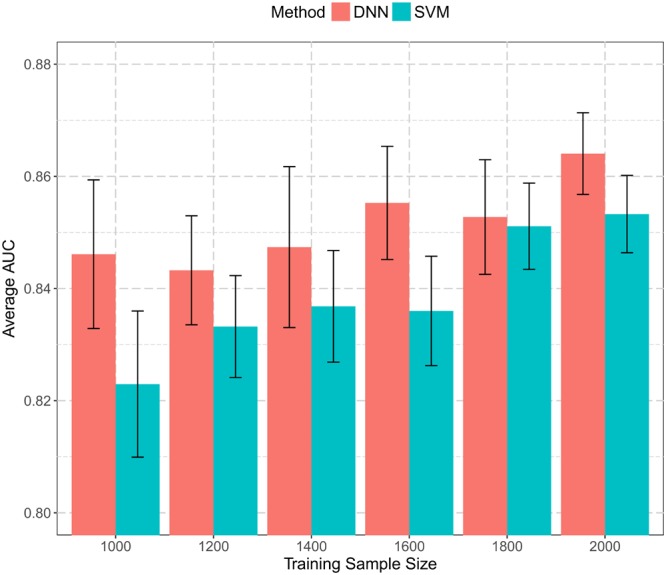
Histogram of prediction performance with varying training sample sizes. A subset of training data with size from 1,000 to 2,000 was extracted and used to build both SVM and DNN models. Average AUCs from 10-fold cross-validation were calculated and plotted.

**Figure 4** summarizes the performance with varying training sample sizes. Both SVM and DNN models achieved relatively good performance. The average AUCs were in the range of 0.82–0.87 in all cases. Even when utilizing only 50% training data, the average AUC still reached 0.823 and 0.846 for SVM and DNN, respectively. The best performance was 0.853 for SVM and 0.864 for DNN, both with all 2,000 proteins used for training. There was a general trend of increasing performance with increasing training sample size for both SVM and DNN models. Also, models trained in the 10-fold cross-validation were more consistent when more proteins were used in the training process. To conclude, the more data we included in classification, the more robust the trained models were. In this work, we used all 2,000 proteins for training, and the sample size we used in this study is probably sufficient.

DNN models generally achieved better performance than SVM models given the same set of proteins for training. Typically, DNN models had many more parameters to be estimated than traditional machine learning methods, and they were more prone to be stuck in local minima. Thus, in order to increase prediction consistency, it is worthwhile to train multiple DNNs under different parameter settings and apply an ensemble strategy to make predictions, which may help improve prediction performance and reduce inconsistencies among all predictors.

### Comparison With Other Tools for General Plant Mitochondrial Targeting Prediction (Independent Testing Set 1)

We further tested the prediction performance between MU-LOC and six existing tools, TargetP (version 1.1), Predotar (version 1.03), MitoProt II (version 1.101), YLoc, MitoFates, and LOCALIZER (version 1.0.2). We used the independent testing set 1 (Supplementary Table S2), which was not in the training process and the maximum sequence identity with training sets was reduced to 40% (see section “Materials and Methods”). This independent testing set contains a mixture of plant mitochondrial proteins with or without N-terminal pre-sequences, reflecting the real-world use case.

**FIGURE 5:**
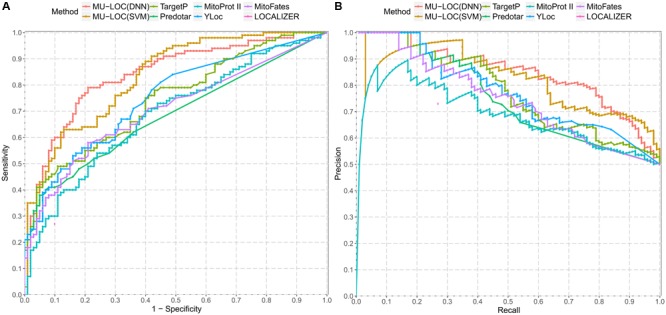
Comparison of prediction performance between MU-LOC and six other tools for general plant mitochondrial targeting prediction (independent testing set 1). **(A)** ROC curves of different methods. “1 – Specificity” is plotted along the *x*-axis. **(B)** Precision–recall curves of different methods. Prediction score of each method on independent testing set 1 was used to calculate and plot corresponding ROC and precision–recall curve. Note that since LOCALIZER does not provide a continuous score for all the testing proteins, we only plotted its performance at the default stringency level (a single point in **A,B**).

The ROC and precision–recall curves of different tools are shown in **Figure 5**, and the performance of each tool under the default parameter settings is summarized in **Table 2**. For MU-LOC, the default parameter is 0.90 estimated specificity level. We used the AUC and F_1_ score to summarize the ROC and precision–recall curves, respectively. First, MU-LOC(DNN) and MU-LOC(SVM) had the highest AUC of 0.8408 and 0.8322. TargetP, Predotar, MitoProt II, YLoc, and MitoFates had AUC of 0.7444, 0.6851, 0.6870, 0.7493, and 0.7050, respectively. Since LOCALIZER did not provide a continuous prediction score for all testing proteins, we did not calculate its AUC. A one-sided bootstrap test using ROCR and pROC R packages indicated a significant higher AUC of MU-LOC(DNN) and MU-LOC(SVM) over TargetP, Predotar, MitoProt II, YLoc, and MitoFates (*p*-value all below 0.05; Sing et al., 2005; Robin et al., 2011). Meanwhile, MU-LOC(DNN) and MU-LOC(SVM) had the highest F_1_ score of 0.698 and 0.675 while the best F_1_ score for other tools was 0.558 (TargetP).

**Table 2:** Performance comparison of MU-LOC with existing tools for general plant mitochondrial targeting prediction (independent testing set 1).

Tool	Parameter	Specificity	Sensitivity	Accuracy	Precision	F_1_ score	MCC
MU-LOC(DNN)	Default	0.88	**0.60**	**0.740**	0.833	**0.698**	**0.500**
MU-LOC(SVM)	Default	0.88	0.57	0.725	0.826	0.675	0.473
TargetP	Default	0.94	0.41	0.675	0.872	0.558	0.413
Predotar	Default	**0.96**	0.33	0.645	**0.892**	0.482	0.373
YLoc	YLoc-LowRes	0.95	0.28	0.615	0.848	0.421	0.310
MitoProt II	Probability > 0.8^a^	0.89	0.33	0.610	0.750	0.458	0.266
MitoFates	Default	**0.96**	0.24	0.600	0.857	0.375	0.288
LOCALIZER	Default	0.90	0.27	0.585	0.730	0.394	0.219

*For the performance metrics used, the higher the value, the better the prediction accuracy. Results with the best performance are highlighted in bold. ^*a*^The webserver of MitoProt II provides only the prediction scores, and we chose a cut-off score of 0.8 to label the prediction class.*

From **Table 2**, MU-LOC has the highest accuracy and MCC under the default parameter setting in all the tools we compared with. MU-LOC(DNN) achieved a sensitivity of 0.60, an accuracy of 0.740, and an MCC of 0.500, while the best performance for other tools (TargetP) had a sensitivity of 0.41, an accuracy of 0.675, and an MCC of 0.413. We listed in Supplementary Table S7 the performance metrics of MU-LOC under different specificity levels. Even under higher specificity levels, MU-LOC still ranked the top in general plant mitochondrial targeting prediction. For instance, under a specificity level of 0.94 (same as TargetP), MU-LOC(DNN) had a sensitivity of 0.49, an accuracy of 0.715, and 0.482, and MU-LOC(SVM) had performance on par with TargetP.

We also investigated the consistency between MU-LOC and other methods using independent testing set 1. Supplementary Figure S2 summarizes the overlap of true positive predictions found by MU-LOC(DNN) and other methods on the independent testing set 1 under the default parameters. MU-LOC(DNN) successfully predicted most of the true positives found by other approaches, covering 91% (52 out of 57), 78% (32 out of 41), 85% (28 out of 33), 82% (27 out of 33), 71% (20 out of 28), 92% (22 out of 24), and 85% (23 out of 27) predictions from MU-LOC(SVM), TargetP, Predotar, MitoProt II, YLoc, MitoFates, and LOCALIZER, respectively. The independent testing proteins and the prediction results by different methods are listed in Supplementary Table S8.

Most existing tools for mitochondrial targeting prediction (TargetP, Predotar, MitoProt II, MitoFates, etc.) focus on using N-terminal sequence information. The high level of consistency between MU-LOC and other methods confirmed the reliability of our method. In addition, MU-LOC has the advantage of predicting mitochondrial proteins lacking N-terminal pre-sequences because we utilized both N-terminal sequence features (AAFreq.NT and PSSM.NT) and global features (PSSM and Coexpr). MU-LOC was able to find more true-positive predictions than other methods without increasing the number of false positives (**Table 2**, Supplementary Table S7, and **Figure 5**).

### Comparison With Other Tools for Predicting Plant Mitochondrial Proteins With N-terminal Pre-sequences (Independent Testing Set 2)

The above independent testing set 1 contains a mixture of mitochondrial proteins with or without N-terminal pre-sequences. Since most of current methods focus on predicting N-terminal targeting sequences, they are effective in using predicted mitochondrial N-terminal pre-sequences. To investigate whether our method captures the features of mitochondrial N-terminal pre-sequences sufficiently without explicitly predicting them, we took advantage of the testing data Sperschneider et al. (2017) used, including 65 plant mitochondrial proteins with N-terminal pre-sequences and 587 non-mitochondrial proteins (Supplementary Table S2). The overlap of this testing set with our training data is minimal, with only six mitochondrial and 16 non-mitochondrial proteins in our training set.

We tested MU-LOC, MitoProt II, and MitoFates on the second independent testing set with the default parameters (Supplementary Table S8). Also, for TargetP, Predotar, YLoc, and LOCALIZER, we directly cited their performances reported by Sperschneider et al. (2017) since the exact same testing set was used (**Table 3**). Both the DNN and SVM models of MU-LOC performed well with accuracy around 94%, and they also had considerably higher specificity, sensitivity, accuracy, and MCC than other tools we compared with. MU-LOC increased the MCC to 0.67 while the best performance for current tools (MitoFates) is around 0.6. Due to the fact that independent testing set 2 was highly imbalanced (65 positive vs. 587 negative), we also calculated the precision (or PPV) in **Table 3** and plotted the precision–recall curve in Supplementary Figure S3A. From the precision–recall curve, MU-LOC performed better than MitoFates, which had the best performance on independent testing set 2 among previous tools that we compared. Also, under the default settings, MU-LOC(DNN) had positive predicted value (PPV) of 0.682, meaning that out of 66 predicted mitochondrial proteins, 45 (68.2%) were true positives (**Table 3**). MU-LOC(SVM) had the highest PPV (0.741) among all methods compared. Supplementary Figure S3B also shows the PPVs of MU-LOC under different cut-offs of prediction scores to estimate the confidence of MU-LOC predictions. MU-LOC(DNN) prediction scores were distributed more uniformly while MU-LOC(SVM) prediction scores were more separated toward the low and high ends. In summary, MU-LOC is at least comparable with the top methods for predicting plant mitochondrial proteins with N-terminal pre-sequences.

**Table 3:** Performance comparison of MU-LOC with existing tools for predicting plant mitochondrial proteins with N-terminal pre-sequences (independent testing set 2).

Tool	Parameter	Specificity	Sensitivity	Accuracy	Precision	MCC
MU-LOC(DNN)	Default	0.964	**0.692**	0.937	0.682	0.652
MU-LOC(SVM)	Default	**0.974**	0.662	**0.943**	**0.741**	**0.669**
TargetP	Sperschneider et al., 2017^b^	0.891	0.646	0.867	0.396	0.440
Predotar	Sperschneider et al., 2017^b^	0.944	0.600	0.910	0.542	0.520
YLoc	Sperschneider et al., 2017^b^	0.940	0.462	0.893	0.462	0.400
MitoProt II	Probability > 0.8^a^	0.842	0.600	0.817	0.295	0.329
MitoFates	Default	0.966	0.615	0.931	0.667	0.602
LOCALIZER	Sperschneider et al., 2017^b^	0.952	0.600	0.917	0.582	0.540

*For the performance metrics used, the higher the value, the better the prediction accuracy. Results with the best performance are highlighted in bold. ^*a*^The webserver of MitoProt II only provides the prediction scores, and we chose a cut-off score of 0.8 to label the prediction class. ^*b*^We cited the performance metrics reported by Sperschneider et al. (2017) since the exact same testing set was used.*

### Predicting Mitochondrial Proteome of *Arabidopsis thaliana* and *Solanum tuberosum*

We applied our MU-LOC(DNN) model to the whole proteome of *A. thaliana* and *Solanum tuberosum*; 27,416 Arabidopsis and 39,011 potato representative protein sequences with at least 50 amino acid residues were downloaded from The Arabidopsis Information Resource (TAIR; Lamesch et al., 2012) and Spud DB^5^, respectively. We utilized the 4,500 negative proteins in the specificity estimation set (see section “Materials and Methods”) to determine the stringency threshold. We increased the default estimated specificity level to limit false-positive predictions. Supplementary Figure S4 summarizes the number of mitochondrial proteins that we predicted for the two plant species under high estimated specificity levels (0.99 and 0.95).

At estimated specificity levels of 0.99 and 0.95, MU-LOC(DNN) predicted 1,031 and 3,205 potential mitochondrial protein candidates in Arabidopsis, respectively (Supplementary Figure S4). At an approximate specificity of 0.95, our predicted 3,205 candidates covered 61% (509 out of 830) Arabidopsis mitochondrial proteins annotated in Swiss-Prot (release 2016_08). This agreement is not surprising since any SwissProt entry was either used in training or shared more than 40% sequence identity with a protein in the training set. Nevertheless, it served as a good consistency check. We also compared our predicted mitochondrial proteome with that from SUBAcon (Hooper et al., 2014). SUBAcon is a Bayesian classifier specifically for Arabidopsis protein localization prediction, which combines 22 computational algorithms, experimental evidence (green fluorescent protein tagging and mass spectrometry), and functional information (protein–protein interaction and gene co-expression). SUBAcon listed 2,671 representative proteins (3,140 isoforms in total) to be located in mitochondria, out of which 1,247 proteins were also predicted by our model.

We also predicted the whole mitochondrial proteome for potato. At an estimated specificity of 0.99 and 0.95, 1,086 and 4,708 proteins were identified as candidates of mitochondrial proteins, respectively (Supplementary Figure S4). Our predictions at the estimated specificity of 0.95 covered 53% (560 out of 1,060) potato mitochondrial proteins currently discovered (Salvato et al., 2014). The predicted Arabidopsis and potato mitochondrial proteomes at a specificity level of 0.95 were summarized in Supplementary Table S9.

We further analyzed functions of our predicted mitochondrial proteome at estimated 95% specificity for *A. thaliana* and *S. tuberosum*, respectively. First, we utilized the gene ontology annotation for Arabidopsis by TAIR (Lamesch et al., 2012; Gene Ontology Consortium, 2015). Among 3,205 predicted mitochondrial protein candidates, 2,741 genes had clear cellular component terms, out of which 1,350 predictions were annotated to be located in mitochondrion (*p*-value < 0.0001 for one-sided Fisher’s exact test compared with genome-wide background distribution of mitochondrial GO annotations). This suggested that approximately half of our predictions were likely to be true. The other half without clear mitochondrial localization information could contain false positives and proteins that are still poorly annotated so far. On the other hand, potato gene annotations are still far from complete. We used a reliable genome-scale annotation, GoMapMan ontology of both Arabidopsis and potato for this study (Ramsak et al., 2014). We found that pentatricopeptide repeat (PPR) proteins annotated in GoMapMan were among the most frequent annotation terms (361 and 625 occurrences in our predicted candidates for Arabidopsis and potato, respectively). Recent studies found that most PPR proteins are located in plant mitochondria and involve in important RNA-linked processes such as RNA editing and RNA splicing (Moller, 2016). However, only 71 PPR proteins have been found in mitochondria by experiments (Salvato et al., 2014; Moller, 2016). Therefore, our predictions may provide useful insight into the involvement of PPR proteins in plant mitochondria. All our predictions at an estimated specificity of 0.95 and their functional annotations were summarized in Supplementary Table S9.

## Discussion

Identifying the proteins belonging to the plant mitochondrial proteome is a crucial step in the study of mitochondrial metabolism. The proteins imported into plant mitochondria play important roles in complex metabolic processes and regulatory mechanisms (Millar et al., 2011; Welchen et al., 2014). Current computational tools have relatively poor performance when applied to this problem (Rao et al., 2016). In this work, we developed MU-LOC, a new approach for the large-scale prediction of plant mitochondrial targeting. After exploring various types of features, we included both sequence-based and functional genomic features in a supervised machine-learning framework, including amino acid composition, protein position weight matrix, and gene co-expression information. These features captured complementary information from different aspects. We then combined all the features and trained a DNN model with the help of GPU computing. Cross-validation and two independent testing results indicate that MU-LOC is robust and outperforms existing tools in predicting mitochondrially localized proteins in plants. When more plant mitochondrial data become available, the improvement of MU-LOC(DNN) over existing tools may be more significant given the nature of DNN. We also applied our DNN models to Arabidopsis and potato proteome, and provided candidate lists of mitochondrial proteins in the two plants. We hope that our predicted mitochondrial proteins in Arabidopsis and potato could be a useful resource for future experiments.

We used both sequence-based and functional features for localization prediction. The sequence-based features were extracted from the N-terminal and the whole protein sequences. The feature selection process indicated that the global PSSM feature from whole sequences contributed the most to the prediction performance. In addition, the gene co-expression feature was demonstrated to be useful for predicting plant mitochondrial targeting. Currently, our model uses only Arabidopsis gene co-expression information. For proteins from other species, our current solution is to use BLAST to find the best Arabidopsis hit. In case we cannot find matches satisfying the e-value cut-off, we assign all zeros to this feature and rely on the sequence-based information to make prediction. For future development, we will include more co-expression data for other plants from public databases such as NCBI GEO (Barrett et al., 2013) and ATTED-II (Aoki et al., 2016). In this way, the co-expression feature will be more accurate and useful. It is worth mentioning that unlike most other prediction methods, neither the global PSSM nor the gene co-expression feature used by MU-LOC requires N-terminal mitochondrial targeting signals. Therefore, compared with other tools, MU-LOC is capable of predicting not only mitochondrial proteins with N-terminal targeting sequences, but also those lacking pre-sequences. In fact, MU-LOC outperforms existing tools in predicting both general mitochondrial targeting and proteins possessing N-terminal pre-sequences in plants, which serves as a practical advantage because for the majority of plant mitochondrial proteins the presence of a pre-sequence is unknown.

We built both DNN and SVM models for the predictions. Both methods achieved good performance on two independent testing sets. SVM outperformed DNN methods slightly on independent testing set 2 (**Table 3**). The positive data in this testing set were plant mitochondrial proteins having N-terminal pre-sequences, representing a small fraction of all proteins localized in mitochondria. In real applications, we may or may not have the N-terminal pre-sequences, as either they do not exist, or no experimental data or reliable prediction is available. Hence, the general plant mitochondrial targeting prediction (with or without N-terminal targeting signals) more reflects real-world problems. DNN performs better than SVM (**Table 2** and **Figure 5**) in this case (independent testing set 1). On the other hand, unlike some other areas such as image processing, we did not observe a dramatic improvement of DNN compared with traditional machine-learning methods. One reason could be that DNN generally needs more data to train and our 2,000 training sample size might still not be big enough. Second, some more advanced DNN architectures such as convolutional neural network (CNN) and RNN may be able to capture the spatial relationships among amino acids better. This will be our future work.

Although MU-LOC showed better prediction performance over existing methods, there are some limitations. Most of the limitations are common to current localization prediction tools, and we will address some of them in our future work. First, no prediction tools can provide tissue- or condition-specific localization predictions. Since the data we collected were from multiple sources under various conditions, our predictions in most cases gave a general probability of a plant protein being localized in mitochondria. Second, we are aware of several new data sources that we have not fully utilized in this work. For example, the updated SUBA4 database was recently released and the cropPAL database contains subcellular localization data for rice, wheat, maize, and barley (Hooper et al., 2016, 2017). We plan to include these new datasets in future versions of our tool. Third, a large number of proteins can be targeted to multiple subcellular compartments. For instance, some plant proteins are imported to both mitochondria and chloroplasts (Peeters and Small, 2001; Carrie and Small, 2013). Accordingly, we can adapt the output layer of our neural networks or integrate other machine-learning algorithms to our framework to handle this multi-label classification problem. Fourth, many plant proteins still lack accurate targeting information. These unlabeled data are not used in our current supervised learning framework. Deep learning has the ability of utilizing unlabeled data in an unsupervised or semi-supervised scenario. Therefore, in the future, we will explore including unlabeled data in our predictive framework. In addition, there is room to explore other types of deep-learning architectures (e.g., CNN and RNN) to further improve the performance. We will also include additional localization data, and expand our methods to other species and more organelles.

## Author Contributions

DX, JT, and IM planned and supervised the project. NZ developed the machine-learning framework, performed the computational experiments, and wrote the manuscript. NZ, RR, FS, and JH collected the plant protein localization data. All the authors contributed to, edited, reviewed, and approved the final version of this manuscript.

## Conflict of Interest Statement

The authors declare that the research was conducted in the absence of any commercial or financial relationships that could be construed as a potential conflict of interest.

## Footnotes

1 http://mu-loc.org/data

2 http://svmlight.joachims.org/

3 http://deeplearning.net/software/theano/

4 http://deeplearning.net/software/pylearn2/

5 http://solanaceae.plantbiology.msu.edu/index.shtml
