# Supplementary material for: MU-LOC: A Machine-Learning Method for Predicting Mitochondrially Localized Proteins in Plants
Source: Front Plant Sci. 2018 May 23;9:634. doi: 10.3389/fpls.2018.00634 (PMC5974146; doi:10.3389/fpls.2018.00634)
Supplement: Supplementary file 3 [file Data_Sheet_1.docx]

**Supplemental Materials**

**Supplementary Methods**

**Meta-Pearson’s Correlation Coefficient (meta-PCC) Calculation**

We followed a meta-analysis approach to obtain the meta-PCC (Srivastava et al., 2010). Firstly, individual Pearson’s correlation coefficient between gene *i* and gene *j* in dataset *k* were calculated and denoted as $r_{ij, k}$.

Next, the individual Pearson’s correlation coefficient was transformed to a standard normal Z score as

$Z_{ij, k}=\frac{1}{2}\log\left( \frac{1+r_{ij, k}}{1-r_{ij, k}} \right)$, (1)

and a weighted average across all the 24 samples was calculated as

$\bar{Z_{ij}}=\frac{\sum_{k=1}^{N} (n_{k}-3)Z_{ij, k}}{\sum_{k=1}^{N} (n_{k}-3)}$, (2)

where *N* is the number of datasets (24 in our case), *n_k_* is the number of samples/experiments in the *k*th dataset. To obtain the meta-PCC between gene *i* and *j* across all datasets, the above weighted average Z score was transformed back using

$r_{ij}^{meta}=\frac{e^{2\bar{Z_{ij}}}-1}{e^{2\bar{Z_{ij}}}+1}$. (3)

Repeat the above process for all pairs of genes to compute the meta-PCC co-expression matrix.

**REFERENCE**

Srivastava, G.P., Qiu, J., and Xu, D. (2010). Genome-wide functional annotation by integrating multiple microarray datasets using meta-analysis. *Int J Data Min Bioinform* 4**,** 357-376.

**Supplementary Tables**

**Supplementary Table S1.** Top 15 Species distribution of proteins collected from Uniprot/Swiss-Prot database.

| Class | Species | Count |
| --- | --- | --- |
| Positive | *Arabidopsis thaliana* | 830 |
|  | *Oryza sativa subsp. japonica* | 118 |
|  | *Solanum tuberosum* | 67 |
|  | *Marchantia polymorpha* | 62 |
|  | *Zea mays* | 40 |
|  | *Oryza sativa subsp. indica* | 31 |
|  | *Pisum sativum* | 23 |
|  | *Prototheca wickerhamii* | 21 |
|  | *Triticum aestivum* | 21 |
|  | *Chlamydomonas reinhardtii* | 19 |
|  | *Oenothera berteroana* | 18 |
|  | *Nicotiana tabacum* | 17 |
|  | *Brassica napus* | 15 |
|  | *Glycine max* | 15 |
|  | *Sorghum bicolor* | 10 |
| Negative | *Arabidopsis thaliana* | 10,144 |
|  | *Oryza sativa subsp. japonica* | 2351 |
|  | *Oryza sativa subsp. Indica* | 595 |
|  | *Zea mays* | 521 |
|  | *Nicotiana tabacum* | 344 |
|  | *Solanum lycopersicum* | 333 |
|  | *Solanum tuberosum* | 279 |
|  | *Pisum sativum* | 261 |
|  | *Chlamydomonas reinhardtii* | 260 |
|  | *Spinacia oleracea* | 256 |
|  | *Glycine max* | 250 |
|  | *Hordeum vulgare* | 224 |
|  | *Triticum aestivum* | 221 |
|  | *Vitis vinifera* | 142 |
|  | *Sorghum bicolor* | 138 |

**Supplementary Table S2.** Training, specificity estimation, and independent testing sets generated from the data collected in this study.

| Type | | Number of proteins |
| --- | --- | --- |
| Training | Positive | 1,000 |
|  | Negative | 1,000 |
| Specificity estimation | Negative | 4,500 |
| Independent testing set 1 | Positive | 100 |
|  | Negative | 100 |
| Independent testing set 2 | Positive | 65 |
|  | Negative | 587 |

**Supplementary Table S3.** GEO datasets used to build co-expression matrix of Arabidopsis genes.

| GEO Accession | Organism | Type | Number of Samples |
| --- | --- | --- | --- |
| GDS416 | *Arabidopsis thaliana* | Count, 2 growth protocol, 3 tissue sets | 11 |
| GDS417 | *Arabidopsis thaliana* | Count, 2 infection, 2 strain sets | 16 |
| GDS453 | *Arabidopsis thaliana* | Transformed count, 5 strain, 4 time sets | 40 |
| GDS515 | *Arabidopsis thaliana* | Count, 3 genotype/variation, 5 protocol sets | 12 |
| GDS672 | *Arabidopsis thaliana* | Count, 3 dose, 2 protocol, 3 time sets | 22 |
| GDS685 | *Arabidopsis thaliana* | Count, 3 genotype/variation, 2 strain sets | 14 |
| GDS1044 | *Arabidopsis thaliana* | Transformed count, 6 agent sets | 21 |
| GDS1466 | *Arabidopsis thaliana* | Transformed count, 6 genotype/variation, 2 tissue sets | 27 |
| GDS1502 | *Arabidopsis thaliana* | Count, 2 genotype/variation, 2 protocol, 4 time sets | 16 |
| GDS1515 | *Arabidopsis thaliana* | Count, 2 agent, 2 genotype/variation, 3 time sets | 14 |
| GDS1620 | *Arabidopsis thaliana* | Count, 17 agent, 2 time sets | 37 |
| GDS1689 | *Arabidopsis thaliana* | Count, 5 growth protocol sets | 15 |
| GDS1723 | *Arabidopsis thaliana* | Count, 2 agent, 2 genotype/variation sets | 12 |
| GDS1727 | *Arabidopsis thaliana* | Transformed count, 3 genotype/variation, 2 stress sets | 15 |
| GDS1757 | *Arabidopsis thaliana* | Count, 2 protocol, 6 time sets | 18 |
| GDS1785 | *Arabidopsis thaliana* | Count, 2 genotype/variation, 3 infection sets | 24 |
| GDS2114 | *Arabidopsis thaliana* | Count, 4 development stage, 3 genotype/variation, 2 tissue sets | 12 |
| GDS2564 | *Arabidopsis thaliana* | Count, 2 genotype/variation, 3 protocol sets | 12 |
| GDS2572 | *Arabidopsis thaliana* | Count, 2 age, 3 genotype/variation sets | 18 |
| GDS3216 | *Arabidopsis thaliana* | Count, 2 stress, 6 time sets | 12 |
| GDS3379 | *Arabidopsis thaliana* | Count, 2 agent, 3 genotype/variation sets | 18 |
| GDS3394 | *Arabidopsis thaliana* | Count, 2 agent, 2 genotype/variation sets | 11 |
| GDS3425 | *Arabidopsis thaliana* | Count, 2 agent, 2 genotype/variation sets | 24 |
| GDS3505 | *Arabidopsis thaliana* | Transformed count, 3 agent, 3 genotype/variation sets | 16 |

**Supplementary Table S4.** Performance measures.

| Metric | Formulas |
| --- | --- |
| Accuracy | $\frac{TP+TN}{TP+FP+FN+TN}$ |
| Specificity | $\frac{TN}{FP+TN}$ |
| Sensitivity | $\frac{TP}{TP+FN}$ |
| Precision (Positive predictive value, PPV) | $\frac{TP}{TP+FP}$ |
| F_1_ score | $\frac{2TP}{2TP+FP+FN}$ |
| Matthews correlation coefficient (MCC) | $\frac{TP\times TN-FP\times FN}{\sqrt{(TP+FP)(TP+FN)(TN+FP)(TN+FN)}}$ |

TP, FP, FN, and TN are the numbers of true positives, false positives, false negatives, and true negatives, respectively.

**Supplementary Table S5.** Performance of features and feature combinations.

| Category | Feature Combination | Average AUC |
| --- | --- | --- |
| N-terminal | AAFreq.NT | 0.721 |
|  | PSSM.NT | 0.775 |
|  | AAFreq.NT + PSSM.NT | 0.776 |
| Whole Sequence | AAFreq | 0.669 |
|  | PSSM | 0.810 |
|  | Coexpr | 0.742 |
|  | PSSM + Coexpr | 0.835 |
|  | AAFreq + PSSM + Coexpr | 0.836 |

AAFreq.NT and PSSM.NT denote amino acid frequency and PSSM feature generated using protein N-terminal sequences, respectively. PSSM and Coexpr denote whole-protein PSSM and the gene co-expression feature, respectively. The average AUCs were calculated using models trained with SVM.

**Supplementary Table S6.** Performance of combinations of features used to build training models.

| Feature | Description | Feature size | Average AUC using only this feature | | Average AUC using all but this feature | |
| --- | --- | --- | --- | --- | --- | --- |
|  |  |  | SVM | DNN | SVM | DNN |
| AAFreq.NT | N-terminal AA frequency | 20 | 0.721 | ─^a^ | 0.850±0.021^c^ | 0.855±0.019^c^ |
| PSSM.NT | N-terminal PSSM | 440 | 0.775 | ─^a^ | 0.852±0.028^c^ | 0.835±0.024^c,d^ |
| PSSM | Whole sequence PSSM | 400 | 0.810 | 0.804 | 0.820±0.026^c,d^ | 0.796±0.030^c,d^ |
| Coexpr | Gene co-expression | 5 | 0.742 | ─^a^ | 0.824±0.028^c,d^ | 0.821±0.024^c,d^ |
| All | All features | 865 | 0.850±0.021^c^ | 0.857±0.022^c^ | ─^b^ | ─^b^ |

^a^ ─, fail to train a working deep neural network in 250 epochs.

^b^ ─, does not apply since all features were used.

^c^, in the format of mean ± standard deviation.

^d^, *p*-value < 0.05 in one sided t-test of testing if the average AUC using all but this feature is less than that using all features.

AAFreq.NT and PSSM.NT denote amino acid frequency and PSSM feature generated using protein N-terminal sequences, respectively. PSSM and Coexpr denote whole-protein PSSM and the gene co-expression feature, respectively.

**Supplementary Table S7.** Performance of MU-LOC under different specificity levels.

| Specificity | MU-LOC | Sensitivity | Accuracy | F1 score | MCC |
| --- | --- | --- | --- | --- | --- |
| 0.99 | SVM | 0.35 | 0.670 | 0.515 | 0.442 |
|  | DNN | 0.21 | 0.600 | 0.344 | 0.320 |
| 0.96 | SVM | 0.39 | 0.675 | 0.545 | 0.426 |
|  | DNN | 0.42 | 0.690 | 0.575 | 0.451 |
| 0.95 | SVM | 0.43 | 0.690 | 0.581 | 0.445 |
|  | DNN | 0.42 | 0.685 | 0.571 | 0.436 |
| 0.94 | SVM | 0.43 | 0.685 | 0.577 | 0.430 |
|  | DNN | 0.49 | 0.715 | 0.632 | 0.482 |
| 0.90 | SVM | 0.56 | 0.730 | 0.675 | 0.489 |
|  | DNN | 0.60 | 0.750 | 0.706 | 0.524 |
| 0.89 | SVM | 0.56 | 0.725 | 0.671 | 0.477 |
|  | DNN | 0.60 | 0.745 | 0.702 | 0.512 |
| 0.80 | SVM | 0.64 | 0.720 | 0.696 | 0.446 |
|  | DNN | 0.77 | 0.785 | 0.782 | 0.570 |

For the performance metrics used, the higher the value, the better the prediction performance.

**Supplementary Table S8.** Supplementary Table S8. List of independent testing data and their prediction scores by different methods under default parameter settings. (**A**) Testing results for general plant mitochondrial targeting prediction. (**B**) Testing results for plant mitochondrial proteins with N-terminal pre-sequences.

**Supplementary Table S9.** Lists of predicted mitochondrial proteins in Arabidopsis and potato at estimated specificity level of 0.95. (**A**) Predicted Arabidopsis mitochondrial proteins with gene ontology annotation. (**B**) Predicted Arabidopsis mitochondrial proteins with GOMapMan annotation. (**C**) Predicted potato mitochondrial proteins with GOMapMan annotation.

**Supplementary Figures**


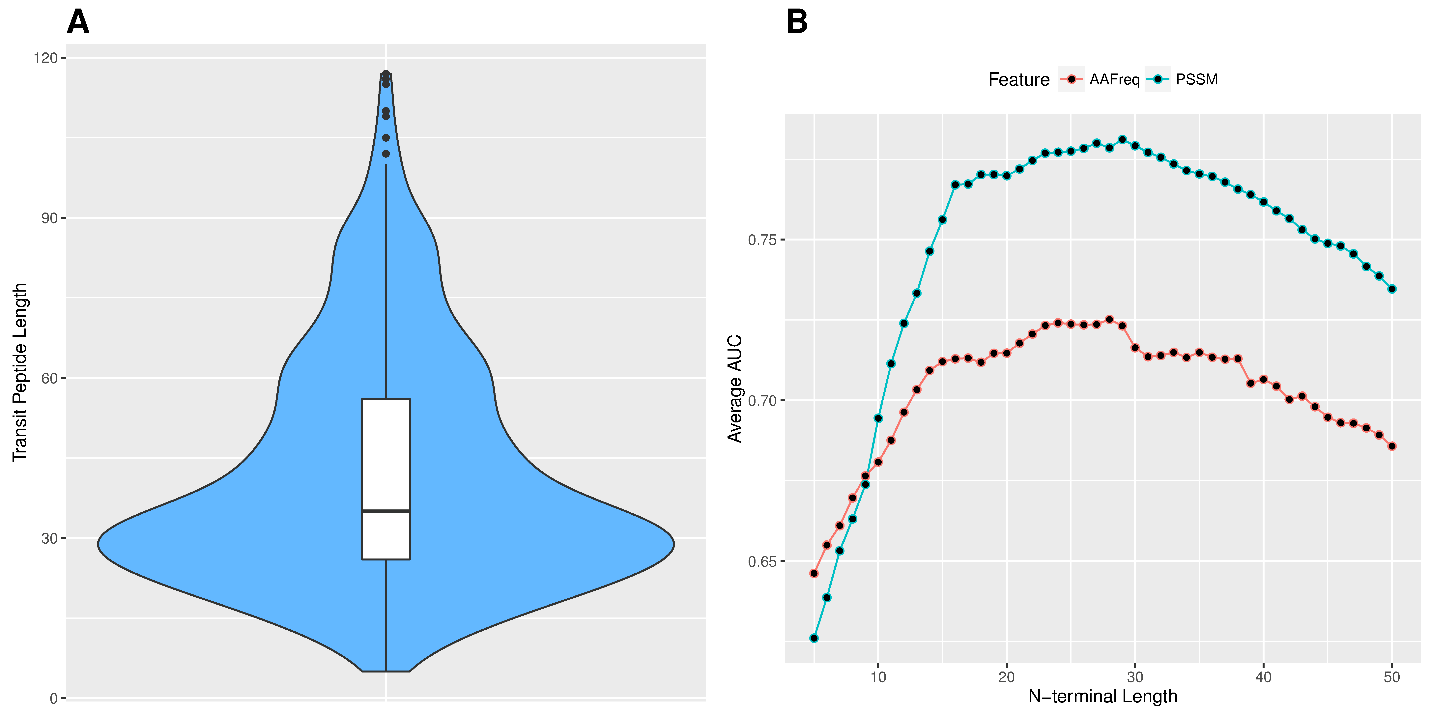


**Supplementary Figure S1.** Performance of N-terminal features with varying N-terminal sequence lengths. (**A**) Violin plot showing the length distribution of 704 annotated plant mitochondrial transit peptides in UniProt/Swiss-Prot (release 2016_08). Some summary statistics are median: 35 amino acids (AA); mean: 42 AA; 70% quantile: 50 AA; and 75% quantile: 56 AA. (**B**) Average AUCs computed from 10-fold cross validation using amino acid frequency and PSSM features generated with N-terminal 5 to 50 residues. SVM is used for the training process.


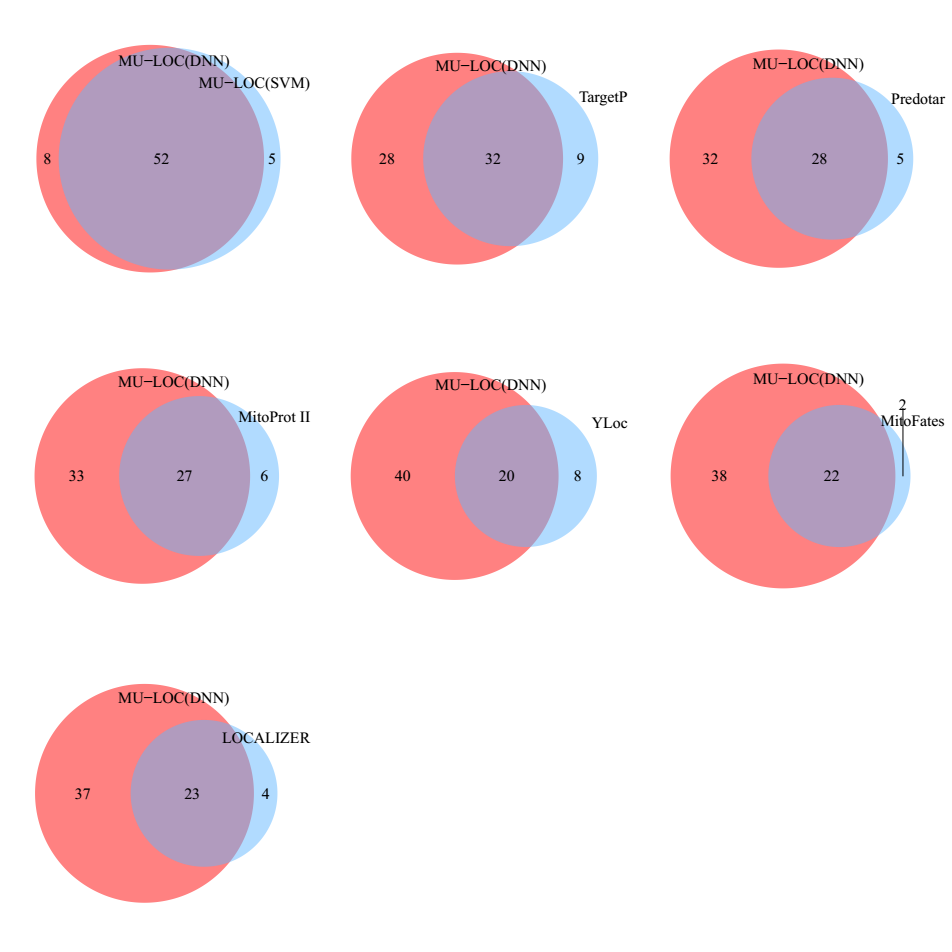


**Supplementary Figure S2.** Venn diagrams of MU-LOC versus other tools on the general plant mitochondrial targeting independent testing set (independent testing set 1). Number of proteins in each area is the true positives predicted by a method under default parameter settings.


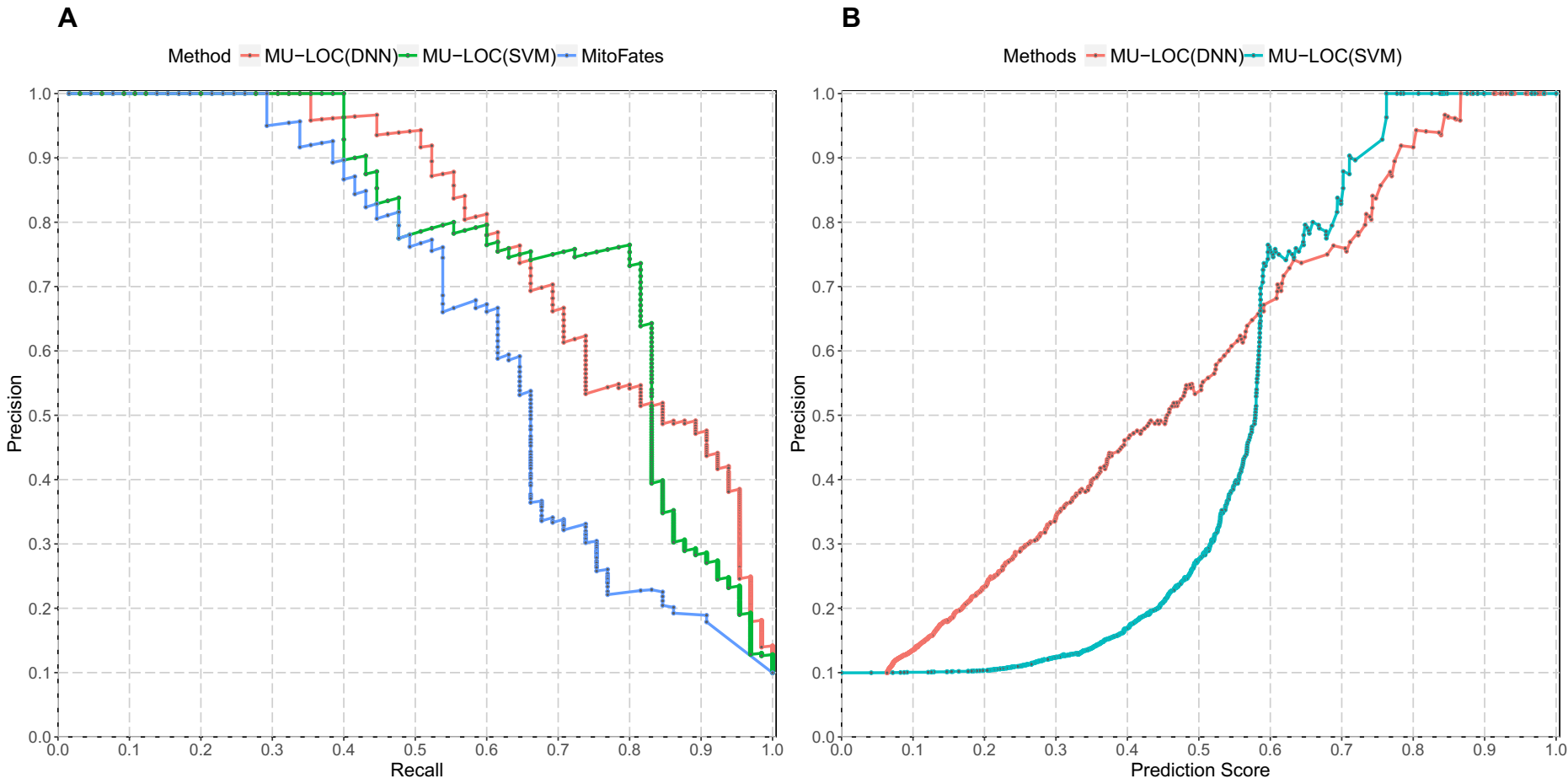


**Supplementary Figure S3.** Performance of MU-LOC on independent testing set 2. (**A**) Precision-recall curves of MU-LOC (DNN and SVM) and MitoFates. MitoFates had the best performance on this testing set among previous tools that we compared. (**B**) Precision or positive predictive value (PPV) calculated using different MU-LOC prediction scores as cutoff. The horizontal axis represents MU-LOC prediction scores and the vertical axis represents the corresponding prediction precision using these scores as cutoff of labeling (mitochondrial or non-mitochondrial). Note that MU-LOC(SVM) prediction scores were min-max scaled to [0, 1].


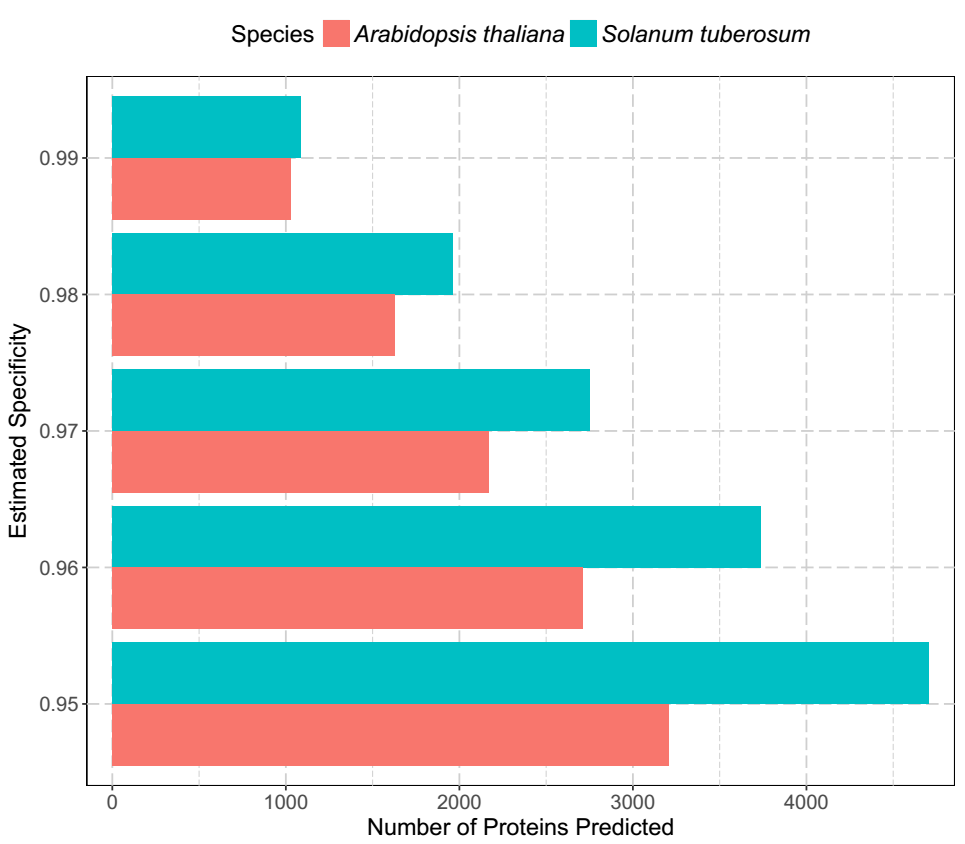


**Supplementary Figure S4.** Number of predicted mitochondrial proteins for Arabidopsis and potato under various estimated specificity levels. The 4,500 negative proteins in the specificity estimation set were used to determine the stringency threshold (specificity).
